# Tracking of Maneuvering Complex Extended Object with Coupled Motion Kinematics and Extension Dynamics Using Range Extent Measurements

**DOI:** 10.3390/s17102184

**Published:** 2017-09-22

**Authors:** Lifan Sun, Baofeng Ji, Jian Lan, Zishu He, Jiexin Pu

**Affiliations:** 1 School of Information Engineering, Henan University of Science and Technology, Luoyang 471023, China; Lifan.sun@gmail.com (L.S.); fengbaoji@126.com (B.J.); pujiexin@126.com (J.P.); 2 School of Electronic Engineering, University of Electronic Science and Technology of China, Chengdu 611731, China; zshe@uestc.edu.cn; 3 Institute of Atmospheric Physics, Chinese Academy of Science, Beijing 100029, China; 4 Center for Information Engineering Science Research (CIESR), School of Electronics and Information Engineering, Xi’an Jiaotong University, Xi’an 710049, China

**Keywords:** maneuvering complex extended object, coupled motion kinematics and extension dynamics, Minkowski sum, range extent measurements

## Abstract

The key to successful maneuvering complex extended object tracking (MCEOT) using range extent measurements provided by high resolution sensors lies in accurate and effective modeling of both the extension dynamics and the centroid kinematics. During object maneuvers, the extension dynamics of an object with a complex shape is highly coupled with the centroid kinematics. However, this difficult but important problem is rarely considered and solved explicitly. In view of this, this paper proposes a general approach to modeling a maneuvering complex extended object based on Minkowski sum, so that the coupled turn maneuvers in both the centroid states and extensions can be described accurately. The new model has a concise and unified form, in which the complex extension dynamics can be simply and jointly characterized by multiple simple sub-objects’ extension dynamics based on Minkowski sum. The proposed maneuvering model fits range extent measurements very well due to its favorable properties. Based on this model, an MCEOT algorithm dealing with motion and extension maneuvers is also derived. Two different cases of the turn maneuvers with known/unknown turn rates are specifically considered. The proposed algorithm which jointly estimates the kinematic state and the object extension can also be easily implemented. Simulation results demonstrate the effectiveness of the proposed modeling and tracking approaches.

## 1. Introduction

In traditional radar- and sonar-based tracking applications, most target tracking approaches usually made the assumption that the received measurement originated from a point source at each time, i.e., a target is often regarded as a point source. Maneuvering target tracking has been extensively studied and well developed in many articles due to its military and civil applications, which has attracted wide attention [1]. However, with the increased resolution capability of modern sensors, an object should be regarded as extended if one target occupies more than one resolution cell or its extent is not negligible compared with the sensor resolution [2,3]. Specifically, some high resolution radars can resolve individual features on an extended object and provide its kinematic measurements. In addition, target detection, classification and tracking require more and more knowledge of the object extension information in practical applications. Thus, treating it as a point mass is no longer reasonable, which results in a significant loss of information. Traditional point tracking algorithms are not suitable for many current practical tracking scenarios. In this context, estimating the kinematic state (i.e., position, velocity, acceleration, etc.) and extension (i.e., size, shape and orientation) of an extended object jointly is referred to as extended object tracking [4]. It has drawn wide attention over the past decades and has been widely applied into the tracking of ground vehicles, close airplanes and ships. Specifically for extended object tracking using (partially) unresolvable measurements of the object’s scattering centers, many models and approaches have been proposed, including the multiple hypothesis tracking (MHT) [5], the spatial probability distribution model [6], and the probability hypothesis density (PHD) filters [7,8,9,10]. A new approach of a random matrix was presented in [11] and improved in [12]. The random-matrix-based approach was also developed to sufficiently characterize time variation and observation distortion [13,14]. In addition, a so-called random hypersurface modeling approach was proposed in [15] to represent the object extensions, as ellipses and star-convex shapes.

Recently, modern and more accurate sensors can also provide the target’s range extent (e.g., down-range and cross-range extent) measurements on a single extended object [16]. Using range extent measurements benefits track retention in practical applications [17]. In [18], recognition of convoys is accomplished by estimating the cross-range extension of the object using target range measurements (high range resolution profiling). For extended object tracking using these types of measurements, several modeling and estimation approaches have also been proposed [19,20,21]. In [22], a new approach based on support functions was proposed to model extended objects. It subsumes the above approaches using range extent measurements and needs no assumption that the orientation of the object is parallel to its velocity vector. However, the support-function-based approach of [22], its variant in [23,24] and other existing approaches have no explicit consideration of extended object’s maneuverability (e.g., turn motions).

This paper aims at solving the maneuvering complex extended object tracking (MCEOT) problem using range extent measurements. Compared with traditional maneuvering target tracking, MCEOT emerges as an important and more difficult problem, i.e., it faces two interrelated challenges in practice: kinematic state and object extension uncertainties. However, the extension dynamics (change in size, shape, orientation, e.g., rotation) of an complex object is not necessarily feasible or convenient to be described and modeled, not to mention it is tightly coupled with the centroid kinematics. In view of the above, how to deal with the MCEOT of both centroid kinematics and the extension dynamics using a target’s range extent is rarely accounted for in existing literature. Actually, different maneuvers of extended objects are always reflected simultaneously in both centroid kinematics and the extension dynamics, e.g., when an extended object maneuvers (e.g., it starts or ends a maneuver), the object motion and extension will undergo an abrupt change.

To sum up, there is a pressing need for solving the MCEOT problem using range extent measurements. Thus, this paper first proposes a motion and extension dynamic model describing constant-turn maneuvers based on Minkowski sum. It can not only describe the complex extension dynamics of an maneuvering complex extended object (MCEO) accurately and effectively, but also fully considers the close coupling between the centroid kinematics and the extension dynamics. Furthermore, this largely facilitates the derivation and design of an MCEOT algorithm for estimating the centroid kinematic state and object extension jointly. Specifically, the proposed algorithm can be easily implemented to deal with different cases of the turn maneuvers with known/unknown turn rates for MCEOT. Compared with existing approaches, our modeling and tracking approach has the following innovative aspects:

(a) MCEOT using measurements of target’s range extent is first considered explicitly, our approach characterizes not only the evolution of the kinematic state over time, but also the object extension dynamics. More importantly, the coupling between the centroid kinematics and extension evolution (e.g., the close relationship between the turn maneuver of the centroid and the abrupt change of extension) is also explicitly involved.
(b) The new model has a concise and unified form and it can accurately describe an MCEO with a turn maneuver in both the extension dynamics and the centroid kinematics, i.e., the maneuver model of a complex extended object can be obtained and directly represented by that of several simple sub-objects (decomposed using the Minkowski sum) jointly. In particular, the elliptical maneuvering object model is obtained in this paper, which is a by-product of the proposed approach.
(c) Based on the Minkowski sum, different parameterizations can be adopted in our unified modeling framework if they are efficient to describe sub-objects’ extension dynamics. This does not affect the generality of the proposed approaches for solving MCEOT.
(d) Due to the concise linear form, the proposed modeling is easily incorporated into a general tracking architecture, in which the exchange of information between centroid kinematics and extension dynamics are sufficiently utilized. This largely facilitates the derivation and design of an MCEOT algorithm for achieving much better estimation performance.

This paper is organized as follows. Section 1 briefly reviews and analyzes the problem existing in the extended object modeling and estimation approach. Section 2 formulates the problem of MCEOT using range extent measurements provided by high resolution sensors. Section 3 proposes a general approach to modeling an MCEO based on Minkowski sum. Different turn maneuvers of a complex extended object (with different turn rates) can be described sufficiently, which has a unified form and is simple to implement. In addition, we propose an MCEO algorithm using the Minkowski-sum-based model for estimating both the kinematic state and extension of an maneuvering extended object, in which different cases of turn maneuvers with known/unknown turn rates for MCEOT are involved and solved explicitly. In Section 4, simulation results are presented to demonstrate the effectiveness of what we propose. The last section concludes the paper.

## 2. Problem Formulation

For a MCEO, different maneuvers are reflected in both centroid kinematics and extension dynamics jointly. However, the complex extension dynamics are usually difficult, infeasible or inconvenient to describe, not to mention it is tightly coupled with the centroid kinematics when the object maneuvers. Thus, this is rarely considered in the existing literature for object tracking using range extent measurements. In this paper, the aim is to estimate the joint state $x_{k}={\left[{\left(x_{k}^{m}\right)}^{T},{\left(x_{k}^{e}\right)}^{T}\right]}^{T}$ of an MCEO using range extent measurements. $x_{k}$ comprises the centroid state $x_{k}^{m}$ ($x_{k}^{m}={\left[x_{k},{\dot{x}}_{k},y_{k},{\dot{y}}_{k}\right]}^{T}$, where (x,y) and $\left(\dot{x},\dot{y}\right)$ are the position and velocity in the two-dimensional (2D) Cartesian coordinate system, respectively) and the vector $x_{k}^{e}$ describing the complex object extension. Consider the following system model:

(1) $$\begin{array}{c} x_{k}=Fx_{k-1}+Gw_{k-1},\\ \;z_{k}=h\left(x_{k},v_{k}\right)\;\;\;\;\;\;\;, \end{array}$$

where $w_{k-1}$ and $v_{k}$ denote the process noise and measurement noise, respectively. The MCEO dynamics of Equation (1) describes the centroid state transition and the change of object extension jointly over time, where *k* is time index. The joint state transition matrix *F* is composed of both the centroid kinematic state transition matrix $F^{m}$ and object extension transition matrix $F^{e}$. The uncertainty of the MCEO state $x_{k}$ is embodied in the process noise $w_{k-1}={\left[{\left(w_{k-1}^{m}\right)}^{T},{\left(w_{k-1}^{e}\right)}^{T}\right]}^{T}.$ Thus, the dynamic equation of Equation (1) has actual the following form:

(2) $$\left[\begin{array}{c} x_{k}^{m}\\ x_{k}^{e} \end{array}\right]=\left[\begin{array}{cc} F^{m} & 0\\ 0 & F^{e} \end{array}\right]\left[\begin{array}{c} x_{k-1}^{m}\\ x_{k-1}^{e} \end{array}\right]+\left[\begin{array}{cc} G^{m} & 0\\ 0 & G^{e} \end{array}\right]w_{k-1}.$$

This paper focuses on the case that an MCEO of interest, assumed to be a rigid body, moves with turn maneuvers in 2D Cartesian coordinate system. As a typical target maneuver, the coordinated turn (CT) motion is also referred to the constant turn (CT), which usually has an approximate constant speed with a constant turn rate [25]. The MCEO dynamics in Equation (2) describes the turn maneuver. Note that different turn maneuvers correspond to different turn rates. In addition, Equation (2) explicitly relates the object extension orientation change to the centroid turn maneuver, which are tightly coupled. The joint transition matrix of the centroid kinematic state and the object extension state is F= diag$(F^{m},F^{e}),$ where

(3) $$F^{m}=\left[\begin{array}{cccc} 1 & \frac{\sin(\omega T)}{\omega } & 0 & -\frac{1-\cos(\omega T)}{\omega }\\ 0 & \cos(\omega T) & 0 & -\sin(\omega T)\\ 0 & \frac{1-\cos(\omega T)}{\omega } & 1 & \frac{\sin(\omega T)}{\omega }\\ 0 & \sin(\omega T) & 0 & \cos(\omega T) \end{array}\right],$$

and ω is the turn rate of the centroid. $F^{m}$ of Equation (3) is a centroid kinematic state transition matrix for modeling of a maneuvering point target with CT motions, which has been extensively researched and applied. According to Equation (3), the centroid state transition of $x_{k}^{m}={\left[x_{k},{\dot{x}}_{k},y_{k},{\dot{y}}_{k}\right]}^{T}$ is

(4) $$x_{k}^{m}=F^{m}x_{k-1}^{m}+G^{m}w_{k-1}^{m},$$

where $G^{m}=$ diag([$\frac{T^{2}}{2},T$]${}^{T},$[$\frac{T^{2}}{2},T$]${}^{T}$), and *T* is the sampling time. Unlike traditional point target tracking, the key to successful MCEOT using range extent measurements lies in the accurate and effective modeling of the object extension dynamics as well as the centroid kinematics.

Specifically for tracking of an extended object using range extent measurements, the support function fits well with the description of extended object extension [22]. Since the support function has natural and intuitive connections to the range extent of an object, the down-range $D(\theta_{k})$ and cross-range extent $C(\theta_{k})$ at viewing angle $\theta_{k}$ can be directly expressed by support functions:

(5) $$\begin{array}{cc} D(\theta_{k}) & =H\left(\theta_{k}\right)+H\left(\theta_{k}+\pi \right), \end{array}$$

(6) $$\begin{array}{cc} C(\theta_{k}) & =H\left(\theta_{k}+\frac{\pi }{2}\right)+H\left(\theta_{k}-\frac{\pi }{2}\right). \end{array}$$

**Figure 1:**
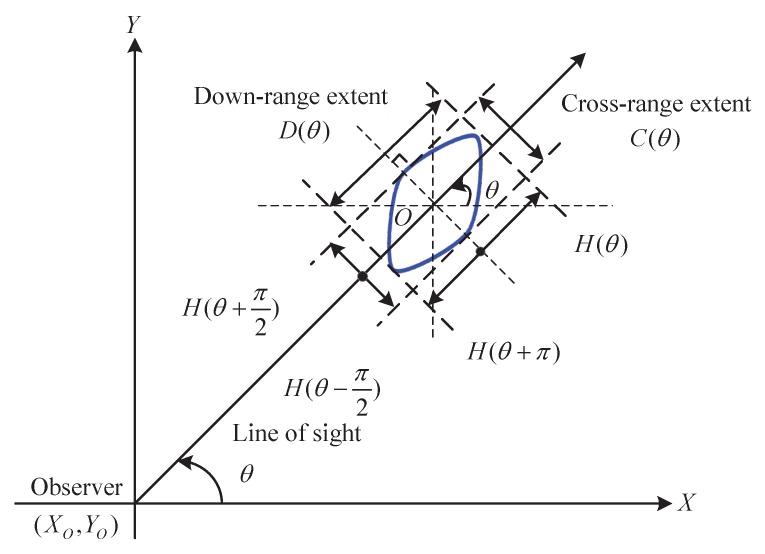
Down-range and cross-range extent.

However, the extension of a somewhat complex extended object (as an example of Figure 1) in some cases is not feasible or convenient to be described by support functions directly using range extent measurements. This certainly brings barriers to the modeling and estimation of the MCEO extension dynamics and there is no explicit consideration in the literature. The major difficulties are summarized as follows:

(a) how to accurately describe the extension dynamics (change in size, shape, orientation, e.g., rotation) of an MCEO over time,
(b) how to deal with the close coupling between the centroid kinematics and extension evolution, and how to embody it in the MCEO modeling.

**Remark** **1.** Compared with traditional maneuvering target tracking, the maneuvering extended object modeling is more difficult and complicated because the extension rotation occurs along with the turn maneuver of the centroid. The close coupling between the centroid maneuver and the change of extension should be considered. Thus, our research on this problem is meaningful and will benefit MCEOT using measurements of range extent, although it is difficult to handle.

## 3. MCEO Modeling Using Range Extent Measurements

### 3.1. The Unified Complex Extension Dynamics Based on Minkowski Sum

**Figure 2:**
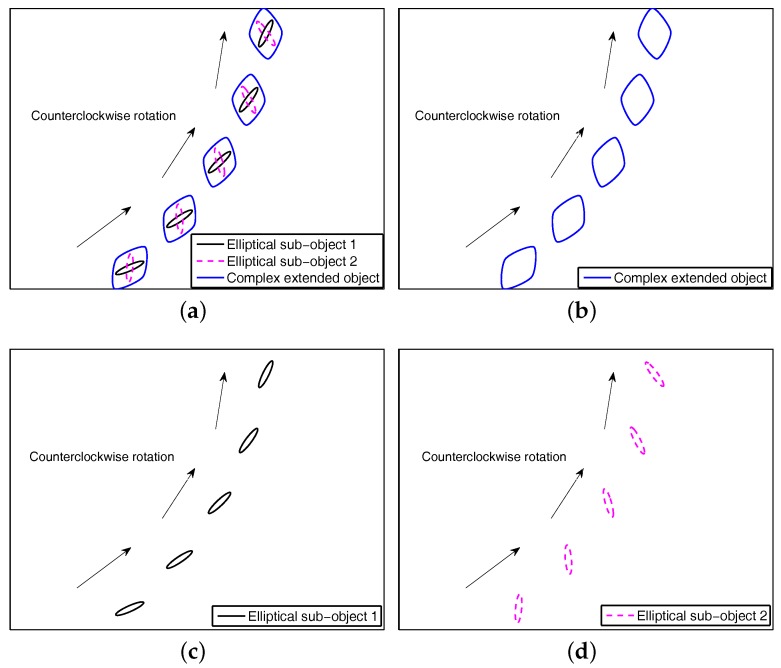
The complex object extension dynamics based on Minkowski sum, (**a**) illustrative example; (**b**) extension dynamics of complex object; (**c**) extension dynamics of sub-object 1; (**d**) extension dynamics of sub-object 2.

In this section, we first consider decomposing an complex extended object as multiple simple sub-objects by Minkowski sum (also known as Minkowski dilation). In this case, the support function representation of the complex object extension can be easily obtained by merging support function representations of these simple sub-object extensions. This is because the sum of support functions is also a support function by using Minkowski sum [23]. Our study finds that the rotation of the complex object extension occurs along with the simultaneous rotation of multiple simple sub-object extensions, i.e., all their rotation motions have exactly the same rotational model with the same turn rate. As examples of an MCEO in Figure 2, its complex extension dynamics in rotation motion at counterclockwise order can be characterized by modeling extension dynamics of sub-objects jointly. In practical applications, the rotation rate of the object extension and the turn rate of the centroid are exactly the same because the extended object is usually treated as a rigid body. Based on the above analysis, the turn maneuver of a complex extended object can be directly modeled by that of several simple sub-objects via Minkowski sum. That is, the MCEO extension dynamics are characterized by multiple simple sub-objects’ extension dynamics jointly, which has a general form as follows:

(7) $$\left[\begin{array}{c} x_{k}^{m}\\ x_{k}^{e,1}\\ \vdots \\ x_{k}^{e,n} \end{array}\right]=\left[\begin{array}{cccc} F^{m} & 0 & 0 & 0\\ 0 & F^{e,1} & 0 & 0\\ 0 & 0 & \ddots & 0\\ 0 & 0 & 0 & F^{e,n} \end{array}\right]\left[\begin{array}{c} x_{k-1}^{m}\\ x_{k-1}^{e,1}\\ \vdots \\ x_{k-1}^{e,n} \end{array}\right]+\left[\begin{array}{cccc} G^{m} & 0 & 0 & 0\\ 0 & G^{e,1} & 0 & 0\\ 0 & 0 & \ddots & 0\\ 0 & 0 & 0 & G^{e,n} \end{array}\right]w_{k-1},$$

where $x_{k-1}^{e,1},\ldots x_{k-1}^{e,n}$ are used to represent each sub-object’s extension, and $F^{e,1}\ldots F^{e,n}$ denote their respective extension transition matrix. In this way, the MCEO extension dynamics modeling is largely simplified because many simple sub-objects’ extension dynamics (e.g., circles, ellipse, rectangles, etc.) are usually available.

In view of the above, the key to the MCEO modeling based on Minkowski sum for tracking is how to obtain effective and concise forms of the sub-objects’ extension dynamics. As an example of Figure 2, these elliptical sub-objects (decomposed by Minkowski sum) are conveniently described by two 2×2 symmetric positive definite matrices. Thus, we have

(8) $$E_{k}^{1}=\left[\begin{array}{cc} E_{k}^{\left(1\right)} & E_{k}^{\left(2\right)}\\ E_{k}^{\left(2\right)} & E_{k}^{\left(3\right)} \end{array}\right],E_{k}^{2}=\left[\begin{array}{cc} E_{k}^{\left(4\right)} & E_{k}^{\left(5\right)}\\ E_{k}^{\left(5\right)} & E_{k}^{\left(6\right)} \end{array}\right].$$

For one elliptical sub-object, its support functions representation $H_{1}\left(\theta_{k}\right)$ at viewing angle $\theta_{k}$ are [22]

(9) $$H_{1}\left(\theta_{k}\right)={\left(v_{k}^{T}\left[\begin{array}{cc} E_{k}^{\left(1\right)} & E_{k}^{\left(2\right)}\\ E_{k}^{\left(2\right)} & E_{k}^{\left(3\right)} \end{array}\right]v_{k}\right)}^{1/2},$$

where $v_{k}={\left[\cos\theta_{k},\sin\theta_{k}\right]}^{T}$ is the unit vector. The Matrix entries of $E_{k}^{1}$ can be included as a parameter vector $x_{k}^{e,1}={\left[E_{k}^{\left(1\right)},E_{k}^{\left(2\right)},E_{k}^{\left(3\right)}\right]}^{T}$ for describing the elliptical extension because they carry useful information about this sub-object’s extension (i.e., its geometric properties are fully reflected by them). Thus, this sub-object’s extension state transition is

(10) $$\begin{array}{c} \;\;\;\;\;x_{k}^{e,1}=x_{k-1}^{e,1}+w_{k-1}^{e,1}\\ ⟹\left[\begin{array}{c} E_{k}^{\left(1\right)}\\ E_{k}^{\left(2\right)}\\ E_{k}^{\left(3\right)} \end{array}\right]=\left[\begin{array}{c} E_{k-1}^{\left(1\right)}\\ E_{k-1}^{\left(2\right)}\\ E_{k-1}^{\left(3\right)} \end{array}\right]+\left[\begin{array}{c} w_{k-1}^{e,(1)}\\ w_{k-1}^{e,(2)}\\ w_{k-1}^{e,(3)} \end{array}\right],\;\; \end{array}$$

where the uncertainty of this object extension is embodied by the process noise $w_{k-1}^{e,1}={\left[w_{k-1}^{e,(1)},w_{k-1}^{e,(2)},w_{k-1}^{e,(3)}\right]}^{T}$. Equation (10) describes the object extension dynamics over time. Actually, this ellipse can be rotated to a different orientation by using $A_{k}$, i.e.,

(11) $$\left[\begin{array}{cc} E_{k}^{\left(1\right)} & E_{k}^{\left(2\right)}\\ E_{k}^{\left(2\right)} & E_{k}^{\left(3\right)} \end{array}\right]=A_{k}\left[\begin{array}{cc} E_{k-1}^{\left(1\right)}+w_{k-1}^{e,(1)} & E_{k-1}^{\left(2\right)}+w_{k-1}^{e,(2)}\\ E_{k-1}^{\left(2\right)}+w_{k-1}^{e,(2)} & E_{k-1}^{\left(3\right)}+w_{k-1}^{e,(3)} \end{array}\right]A_{k}^{T},$$

which describes the change of extension orientation over time. In a 2D space, every rotation matrix is

(12) $$A_{k}=\left[\begin{array}{cc} \cos\phi & -\sin\phi \\ \sin\phi & \cos\phi \end{array}\right].$$

Substituting Equation (12) into Equation (11) yields

(13) $$\begin{array}{cc} \left[\begin{array}{c} E_{k}^{\left(1\right)}\\ E_{k}^{\left(2\right)}\\ E_{k}^{\left(3\right)} \end{array}\right] & =\left[\begin{array}{ccc} \cos^{2}\phi & -\sin2\phi & \sin^{2}\phi \\ \frac{1}{2}\sin2\phi & \cos2\phi & -\frac{1}{2}\sin2\phi \\ \sin^{2}\phi & \sin2\phi & \cos^{2}\phi \end{array}\right]\left[\begin{array}{c} E_{k-1}^{\left(1\right)}\\ E_{k-1}^{\left(2\right)}\\ E_{k-1}^{\left(3\right)} \end{array}\right]\\ & +\left[\begin{array}{ccc} \cos^{2}\phi & -\sin2\phi & \sin^{2}\phi \\ \frac{1}{2}\sin2\phi & \cos2\phi & -\frac{1}{2}\sin2\phi \\ \sin^{2}\phi & \sin2\phi & \cos^{2}\phi \end{array}\right]\left[\begin{array}{c} w_{k-1}^{e,(1)}\\ w_{k-1}^{e,(2)}\\ w_{k-1}^{e,(3)} \end{array}\right]\\ & ⟹x_{k}^{e,1}=F^{e,1}x_{k-1}^{e,1}+G^{e,1}w_{k-1}^{e,1}, \end{array}$$

where ϕ=ωT is the rotation angle in counterclockwise order, and

(14) $$F^{e,1}=G^{e,1}=\left[\begin{array}{ccc} \cos^{2}\omega T & -\sin2\omega T & \sin^{2}\omega T\\ \frac{1}{2}\sin2\omega T & \cos2\omega T & -\frac{1}{2}\sin2\omega T\\ \sin^{2}\omega T & \sin2\omega T & \cos^{2}\omega T \end{array}\right].$$

For the other elliptical object in Figure 2, its support functions representation $H_{2}\left(\theta_{k}\right)$ at viewing angle $\theta_{k}$ is

(15) $$H_{2}\left(\theta_{k}\right)={\left(v_{k}^{T}\left[\begin{array}{cc} E_{k}^{\left(4\right)} & E_{k}^{\left(5\right)}\\ E_{k}^{\left(5\right)} & E_{k}^{\left(6\right)} \end{array}\right]v_{k}\right)}^{1/2}.$$

These matrix entries $E_{k}^{\left(4\right)},E_{k}^{\left(5\right)},E_{k}^{\left(6\right)}$ can be taken as its extension parameters $x_{k}^{e,2}={\left[E_{k}^{\left(4\right)},E_{k}^{\left(5\right)},E_{k}^{\left(6\right)}\right]}^{T}$. Since the rotation motion of two sub-objects have exactly the same rotation mode with the same turn rate, the sub-object extension transition of $x_{k}^{e,2}$ is

(16) $$x_{k}^{e,2}=F^{e,1}x_{k-1}^{e,2}+G^{e,1}w_{k-1}^{e,2},$$

where $w_{k-1}^{e,2}={\left[w_{k-1}^{e,(4)},w_{k-1}^{e,(5)},w_{k-1}^{e,(6)}\right]}^{T}$. As mentioned before, the extension dynamics of this complex extended object in rotation motion can be characterized by modeling extension dynamics of sub-objects jointly from Equations (13) and (16), i.e.,

(17) $$\begin{array}{cc} \left[\begin{array}{c} x_{k}^{e,1}\\ x_{k}^{e,2} \end{array}\right] & =\left[\begin{array}{cc} F^{e,1} & 0\\ 0 & F^{e,1} \end{array}\right]\left[\begin{array}{c} x_{k-1}^{e,1}\\ x_{k-1}^{e,2} \end{array}\right]+\left[\begin{array}{cc} G^{e,1} & 0\\ 0 & G^{e,1} \end{array}\right]\left[\begin{array}{c} w_{k-1}^{e,1}\\ w_{k-1}^{e,2} \end{array}\right],\\ x_{k}^{e} & =F^{e}x_{k-1}^{e}+G^{e}w_{k-1}^{e}, \end{array}$$

where $F^{e}=G^{e}=$diag$(F^{e,1},F^{e,1})=$diag$\left(G^{e,1},G^{e,1}\right)$. These elliptical sub-object have the same extension evolution over time for describing the turn maneuver, which facilitates modeling of the complex object extension dynamics for tracking.

Note that other complex object extensions can also be described as the Minkowski sums of other simple sub-objects with different extensions (besides ellipses) represented by support functions. The elliptical maneuvering object model is a by-product and actually considered as a special case of the proposed approach based on Minkowski sum.

**Remark** **2.** *$F^{e}=G^{e}=$ diag(1,1,1,1,1,1) in Equation (17) when the turn rate ω=0. Correspondingly, the centroid kinematic state transition matrix of the CT motion in Equation (3) is also changed to $F^{m}=$ diag$\left(F^{CV},F^{CV}\right)$, where $F^{CV}=\left[\begin{array}{cc} 1 & T\\ 0 & 1 \end{array}\right].$ The centroid state transition in Equation (4) is rewritten by*

(18) $$x_{k}^{m}=\mathrm{diag}\left(F^{CV},F^{CV}\right)x_{k-1}^{m}+G^{m}w_{k-1}^{m}.$$

In this case, the proposed maneuvering extended object model for MCEOT (e.g., describing constant turn motion) reduces to the non-maneuvering object model (e.g., describing constant velocity motion) when the turn rate ω=0. Given more information, we can design more specifically for different MCEOT scenarios because different $F^{m}$ and $F^{e}$ result in different centroid state and object extension transition, respectively.

As mentioned above, there is a close coupling between the centroid maneuver and the change of extension, i.e., the extension rotation occurs along with the turn maneuver of the centroid. Thus, the rotation rate of extension $\omega =\frac{\phi }{T}$ in $A_{k}$ (see Eqaution (12)) and the turn rate ω of the centroid (see Eqaution (3)) are exactly the same when an extended object maneuvers (e.g., performing a turn motion). The transition of $x_{k}^{e}$ in Equation (17) is actually the object extension dynamic model, which describes the change of the extension orientation over time. Equation (7) also explicitly describes the relation between the object extension orientation change and the centroid turn maneuver. The extension state $x_{k}^{e}={\left[{\left(x_{k}^{e,1}\right)}^{T},{\left(x_{k}^{e,2}\right)}^{T}\right]}^{T}$ is included in $x_{k}={\left[x_{k},{\dot{x}}_{k},y_{k},{\dot{y}}_{k},E_{k}^{\left(1\right)},E_{k}^{\left(2\right)},E_{k}^{\left(3\right)},E_{k}^{\left(4\right)},E_{k}^{\left(5\right)},E_{k}^{\left(6\right)}\right]}^{T}$ as the joint state vector. Note that different $F^{m}$ and $F^{e}$ describe different transition of centroid kinematic state and the change of object extension orientation, respectively.

**Remark** **3.** The MCEO modeling approach based on Minkowski sum using range extent measurements has several following advantages: (1) It has a concise mathematical form to describe an MCEO with different turn motions accurately; (2) the close relationship between the maneuver of the centroid and the change of object extension orientation is considered and solved explicitly; (3) the proposed modeling approach is easy to implement and facilitates the derivation and design of MCEOT algorithms; and (4) the matrix parameterization is not the only option for our approach to solve the MCEOT using target’s range extent measurements. Other parameterizations can also be adopted in the unified model framework based on Minkowski sum if they are efficient to describe other convex objects. This does not affect the generality of the proposed approaches.

For tracking of an MCEO (e.g., a maneuvering civil or military aircraft) that performs CT motions with known/unknown turn rates, we propose two different Minkowski-sum-based MCEOT algorithms for estimating the joint target state $x_{k}$ (i.e., both the centroid kinematic state $x_{k}^{m}$ and the extension state $x_{k}^{e}$) in this section.

### 3.2. The Minkowski-Sum-Based Modeling and Estimation for CT Maneuvers with Known Turn Rates

In the first case of constant turn maneuvers with known turn rates, we introduce a hybrid system framework for describing practical motions and extension dynamics of an extended object accurately. It is beneficial to use more than one MCEO motion model with known turn rates in a tracking algorithm when the true object motion is complicated, e.g., the whole process of turn motions is assumed to be described by a model set (e.g., including a non-maneuvering model and several turn maneuver models). Thus, we consider the following hybrid system:

(19) $$\begin{array}{c} \;\;\;\;\;x_{k}=F^{\left(i\right)}x_{k-1}+w_{k-1}^{\left(i\right)},\;w_{k-1}^{\left(i\right)}∼N\left(0,Q_{k}^{\left(i\right)}\right)\\ z_{k}=h\left(x_{k},v_{k}\right),\;v_{k}∼N\left(0,R_{k}\right),\;\;\; \end{array}$$

where superscript *i* denotes quantities pertinent to model $m^{\left(i\right)}$ in the set $M=\{m^{\left(1\right)},m^{\left(2\right)},\ldots ,m^{\left(M\right)}\}$. The modal state $m_{k}$ sequence is a first-order Markov chain that has transition probabilities $\pi_{i,j}=P\left\{m_{k}^{\left(j\right)}|m_{k-1}^{\left(i\right)}\right\}$, $\forall m^{\left(i\right)}$, $m^{\left(j\right)}$, *k*. $m_{k}^{\left(i\right)}$ means that model $m^{\left(i\right)}$ matching the system motion mode (e.g., turn motions with different turn rates) is in effect at time k.

Similar to [17,20], we also assume that a high resolution radar provides target range extent measurements (i.e., $D(\theta_{k})$ and $C(\theta_{k})$) as well as the range $r_{k}$ and bearing $\beta_{k}$ measurements of the object centroid. Thus, the measurement equation is written as

(20) $$z_{k}={\left[r_{k},\beta_{k},D_{k},C_{k}\right]}^{T}+v_{k},$$

where $r_{k}=\sqrt{\left(x_{k}-X_{0}\right){}^{2}+\left(y_{k}-Y_{0}\right){}^{2}}$, $\beta_{k}=$ arctan$\frac{\left(y_{k}-Y_{0}\right)}{\left(x_{k}-X_{0}\right)}$ and $v_{k}$ is measurement noise. $\left(X_{0},Y_{0}\right)$ is the location of the sensor. The measurements in z${}_{k}={\left[r_{k},\beta_{k},D_{k},C_{k}\right]}^{T}$ are usually provided from different physical channels, and the noise $v_{k}$ is generally assumed to be a zero-mean Gaussian process with cov$\left[v_{k}\right]=R_{k}=$diag$\left[R_{k}^{r},R_{k}^{\beta },R_{k}^{D},R_{k}^{C}\right]$. As an example of a complex object in Figure 1, this object can be described as a Minkowski sum of two elliptical sub-objects, and its support function representation has the following form: $H\left(\theta_{k}\right)=H_{1}\left(\theta_{k}\right)+H_{2}\left(\theta_{k}\right).$ Thus, Equations (5) and (6) are rewritten as

(21) $$D\left(\theta_{k}\right)=H\left(\theta_{k}\right)+H\left(\theta_{k}+\pi \right)=H_{1}\left(\theta_{k}\right)+H_{2}\left(\theta_{k}\right)+H_{1}\left(\theta_{k}+\pi \right)+H_{2}\left(\theta_{k}+\pi \right),$$

(22) $$C\left(\theta_{k}\right)=H\left(\theta_{k}+\frac{\pi }{2}\right)+H\left(\theta_{k}-\frac{\pi }{2}\right)=H_{1}\left(\theta_{k}+\frac{\pi }{2}\right)+H_{2}\left(\theta_{k}+\frac{\pi }{2}\right)+H_{1}\left(\theta_{k}-\frac{\pi }{2}\right)+H_{2}\left(\theta_{k}-\frac{\pi }{2}\right).$$

Since the elliptical sub-objects are centrosymmetric, i.e.,

(23) $$H\left(\theta_{k}\right)=H\left(\theta_{k}+\pi \right),H\left(\theta_{k}+\frac{\pi }{2}\right)=H\left(\theta_{k}-\frac{\pi }{2}\right),$$

we have

(24) $$D\left(\theta_{k}\right)=2H\left(\theta_{k}\right)=2\left(H_{1}\left(\theta_{k}\right)+H_{2}\left(\theta_{k}\right)\right),$$

(25) $$C\left(\theta_{k}\right)=2H\left(\theta_{k}+\frac{\pi }{2}\right)=2\left(H_{1}\left(\theta_{k}+\frac{\pi }{2}\right)+H_{2}\left(\theta_{k}+\frac{\pi }{2}\right)\right).$$

For this case of turn maneuvers with known turn rates, we propose an MCEOT algorithm within the multiple model framework [26] by comprehensively considering the uncertainties of motion and extension dynamics. It runs a set of filters based on models describing several possible turn maneuvers as well as the non-maneuver target motion. Suppose that the required state estimate ${\hat{x}}_{k-1|k-1}^{\left(i\right)}={\left[{\hat{x}}_{k-1|k-1}^{m,(i)},{\hat{x}}_{k-1|k-1}^{e,(i)}\right]}^{T},$ its error covariance ${\bar{P}}_{k-1|k-1}^{\left(i\right)}$ and model probability $\mu_{k-1}^{\left(i\right)}$ at time k−1 are available for $m^{\left(i\right)},i=1,\ldots ,N$. ${\hat{x}}_{k|k}$ and $P_{k|k}$ can be obtained recursively (k−1→k) in prediction and updated as follows.

Step 1. Evaluate the mixing probabilities $\mu_{k-1}^{j|i}=$$\pi_{j,i}\mu_{k-1}^{\left(j\right)}/\mu_{k|k-1}^{\left(i\right)}$ with $\mu_{k|k-1}^{\left(i\right)}=\sum_{j=1}^{M}\pi_{j,i}\mu_{k-1}^{\left(j\right)}$, and mixing estimates ${\bar{x}}_{k-1|k-1}^{\left(i\right)}=\sum_{j=1}^{M}{\hat{x}}_{k-1|k-1}^{\left(j\right)}\mu_{k-1}^{j|i}$ and covariance ${\bar{P}}_{k-1|k-1}^{\left(i\right)}=\sum_{j=1}^{M}\left[P_{k-1|k-1}^{\left(j\right)}+\left({\bar{x}}_{k-1|k-1}^{\left(i\right)}-{\hat{x}}_{k-1|k-1}^{\left(i\right)}\right){\left({\bar{x}}_{k-1|k-1}^{\left(i\right)}-{\hat{x}}_{k-1|k-1}^{\left(i\right)}\right)}^{T}\right]\mu_{k-1}^{j|i}$.

Step 2. Run a nonlinear filter (e.g., unscented filter, extended Kalman filter, quadrature Kalman filter, etc.) for each model with initial condition (${\hat{x}}_{k-1|k-1}^{\left(i\right)}={\left[{\hat{x}}_{k-1|k-1}^{m,(i)},{\hat{e}}_{k-1|k-1}^{\left(i\right)}\right]}^{T}$, $P_{k-1|k-1}^{\left(i\right)}$ and $\mu_{k-1}^{\left(i\right)}$).

Step 3. Model probability update is derived by Bayes’ formula, i.e., $\mu_{k}^{\left(i\right)}=\frac{\Lambda_{k}^{\left(i\right)}\mu_{k|k-1}^{\left(i\right)}}{\sum_{j=1}^{N}\Lambda_{k}^{\left(j\right)}\mu_{k|k-1}^{\left(i\right)}}$, where $\Lambda_{k}^{\left(i\right)}=p\left[z_{k}|z^{k-1},m_{k}^{\left(i\right)}\right]$ is the likelihood of model $m_{k}^{\left(i\right)}$. It is usually approximated as a Gaussian distribution by moment matching, i.e $\Lambda_{k}^{\left(i\right)}=N\left(z_{k}-{\hat{z}}_{k|k-1}^{\left(i\right)};0,S_{k}^{\left(i\right)}\right)$.

Step 4. The fused kinematic state and extension state estimate is calculated as the sum of ${\hat{x}}_{k|k}^{\left(i\right)}={\left[{\hat{x}}_{k|k}^{m,(i)},{\hat{e}}_{k|k}^{\left(i\right)}\right]}^{T}$ weighted by its corresponding model probabilities $\mu_{k}^{\left(i\right)}$: ${\hat{x}}_{k|k}=\sum_{i=1}^{N}{\hat{x}}_{k|k}^{\left(i\right)}\mu_{k}^{\left(i\right)}$ and $P_{k|k}=\sum_{i=1}^{N}\left[P_{k|k}^{\left(i\right)}+\left({\hat{x}}_{k|k}-{\hat{x}}_{k|k}^{\left(i\right)}\right){\left({\hat{x}}_{k|k}-{\hat{x}}_{k|k}^{\left(i\right)}\right)}^{T}\right]\mu_{k}^{\left(i\right)}$.

**Remark** **4.** Thanks to the concise linear form of the Minkowski-sum-based dynamic model given the known turn rate, the uncertainty in turn rate can be alleviated by the proposed algorithm. However, turn maneuvers are not necessarily totally covered by several CT models with known turn rates—not to say the true turn rate is usually unknown for the tracker in practical applications. This case can be handled by another modeling and estimation algorithm proposed next.

### 3.3. The Minkowski-Sum-Based Modeling and Estimation for CT Maneuvers with Unknown Turn Rates

For tracking of an MCEO that performs CT motions with unknown turn rates, we propose another Minkowski-sum-based MCEO modeling and estimation algorithm. In many CT maneuver cases, the turn rate is usually unknown or not be known a priori for the tracker. Thus, different from the case of constant turn maneuvers with known turn rates, it can be described by the Wiener process:

(26) $$\omega_{k}=\omega_{k-1}+w_{k-1}^{\omega }.$$

where $w_{k-1}^{\omega }$ is zero-mean Gaussian noise. In this case, the unknown turn rate is included as a state component into the joint state vector, to be estimated recursively. That is, we augment the object state vector $x_{k}={\left[{\left(x_{k}^{m}\right)}^{T},{\left(x_{k}^{e}\right)}^{T}\right]}^{T}$ to include it:

(27) $$x_{k}^{A}≜{\left[{\left(x_{k}\right)}^{T},\omega_{k}\right]}^{T}.$$

The MEO dynamic model of this case is reformulated by Equations (2) and (26) jointly, i.e.,

(28) $$\begin{array}{c} x_{k}^{A}=F_{k-1}^{A}x_{k-1}^{A}+G_{k-1}^{A}w_{k-1}^{A}\\ \left[\begin{array}{c} x_{k}^{m}\\ x_{k}^{e}\\ \omega_{k} \end{array}\right]=\left[\begin{array}{ccc} F_{k-1}^{m} & 0 & 0\\ 0 & F_{k-1}^{e} & 0\\ 0 & 0 & 1 \end{array}\right]\left[\begin{array}{c} x_{k-1}^{m}\\ x_{k-1}^{e}\\ \omega_{k-1} \end{array}\right]+\left[\begin{array}{ccc} G_{k-1}^{m} & 0 & 0\\ 0 & G_{k-1}^{e} & 0\\ 0 & 0 & 1 \end{array}\right]\left[\begin{array}{c} w_{k-1}^{m}\\ w_{k-1}^{e}\\ w_{k-1}^{\omega } \end{array}\right], \end{array}$$

where

(29) $$F_{k-1}^{m}=\left[\begin{array}{cccc} 1 & \frac{\sin(\omega_{k-1}T)}{\omega_{k-1}} & 0 & -\frac{1-\cos(\omega_{k-1}T)}{\omega_{k-1}}\\ 0 & \cos(\omega_{k-1}T) & 0 & -\sin(\omega_{k-1}T)\\ 0 & \frac{1-\cos(\omega_{k-1}T)}{\omega_{k-1}} & 1 & \frac{\sin(\omega_{k-1}T)}{\omega_{k-1}}\\ 0 & \sin(\omega_{k-1}T) & 0 & \cos(\omega_{k-1}T) \end{array}\right],$$

and $F_{k-1}^{e}=G_{k-1}^{e}=$diag$(F_{k-1}^{e,1},F_{k-1}^{e,1}),$

(30) $$F_{k-1}^{e,1}=\left[\begin{array}{ccc} \cos^{2}\omega_{k-1}T & -\sin2\omega_{k-1}T & \sin^{2}\omega_{k-1}T\\ \frac{1}{2}\sin2\omega_{k-1}T & \cos2\omega_{k-1}T & -\frac{1}{2}\sin2\omega_{k-1}T\\ \sin^{2}\omega_{k-1}T & \sin2\omega_{k-1}T & \cos^{2}\omega_{k-1}T \end{array}\right].$$

Thus, the following dynamic and measurement equations of the system are considered:

(31) $$\begin{array}{c} \;\;\;\;x_{k}^{A}=F_{k-1}^{A}x_{k-1}^{A}+G_{k-1}^{A}w_{k-1}^{A},\;w_{k-1}^{A}∼N\left(0,Q_{k}^{A}\right)\\ z_{k}=h\left(x_{k},v_{k}\right),\;v_{k}∼N\left(0,R_{k}\right)\;\;\;. \end{array}$$

Note that $F_{k-1}^{A}=$diag$\left(F_{k-1}^{m},F_{k-1}^{e},1\right)$ in Equation (31) is totally different from F=diag$\left(F^{m},F^{e}\right)$ in Equation (19) because here the turn rate $\omega_{k-1}$ is considered as an unknown state component. In this case, the dynamic model of Equation (28) is really no longer linear, and a nonlinear tracking algorithm needs to be designed with a joint state vector $x_{k}^{A}$ (including the unknown turn rate). The measurement equation of Equation (31) is still the same as those of Equation (19). Suppose that augmented state estimate ${\hat{x}}_{k-1|k-1}^{A}={\left[{\left({\hat{x}}_{k-1|k-1}^{A}\right)}^{T},\omega_{k}\right]}^{T}$ and its error covariance $P_{k-1|k-1}^{A}$ at time k−1 are available, we adopt the unscented transformation (UT) [27] to solve the severely nonlinear dynamic and measurement equations, i.e.,

(32) $$\begin{array}{c} \;\;\;\;\left({\hat{x}}_{k|k-1}^{A},P_{k|k-1}^{A}\right)=\mathrm{UT}\left[F_{k-1}^{A}x_{k-1}^{A},{\left({\hat{x}}_{k-1|k-1}^{A}\right)}^{T},P_{k-1|k-1}^{A}\right],\\ \left({\hat{z}}_{k|k-1},S_{k}\right)=\mathrm{UT}\left[h\left(x_{k}^{A}\right),{\left({\hat{x}}_{k|k-1}^{A}\right)}^{T},P_{k|k-1}^{A}\right].\; \end{array}$$

The UT is an effective approach to approximating first and second moments (e.g., mean and covariance) as a nonlinear function of random state vectors $x_{k}^{A}$ and $z_{k}$ by deterministic sample points with weights $\left\{\alpha^{i},i=0,1,\ldots ,N\right\}$. Note that the UT is not the only option; other moment approximating methods (e.g., the uncorrelated conversion [28,29], the Gauss–Hermite quadrature rules [30], the Cubature rules [31], etc.) may also be used here. Excluding Equation (32), the remaining estimate process of Equation (31) can be implemented in the framework of Kalman filter directly due to the proposed concise model describing CT maneuvers with unknown turn rates.

### 3.4. Complexity Analysis

In our modeling and tracking framework, a complex object is decomposed as multiple simple sub-objects (i.e., $K_{1},K_{2},\ldots K_{N_{s}}$) by using the Minkowski sum. If they are represented by support functions (i.e., $H_{K_{1}}\left(\theta \right),H_{K_{2}}\left(\theta \right),\ldots ,H_{K_{N_{s}}}\left(\theta \right)$) for the viewing angle θ, the Minkowski sum $K_{1}⊕K_{2}⊕,\ldots ,⊕K_{N_{s}}$ is unique with

(33) $$H_{K_{1}⊕K_{2}⊕,\ldots ,⊕K_{N_{s}}}\left(\theta \right)=H_{K_{1}}\left(\theta \right)+H_{K_{2}}\left(\theta \right)+\ldots ,+H_{K_{N_{s}}}\left(\theta \right),$$

where $H_{K_{1}}\left(\theta \right)+H_{K_{2}}\left(\theta \right)+\ldots ,+H_{K_{N_{s}}}\left(\theta \right)$ is also a support function. As an example of Figure 2, the Minkowski sum of a complex object (i.e., $K_{1}⊕K_{2}$) becomes $H_{K_{1}⊕K_{2}}\left(\theta \right)=H_{K_{1}}\left(\theta \right)+H_{K_{2}}\left(\theta \right)$. Correspondingly, its extension state $x_{k}^{e}={\left[E_{k}^{\left(1\right)},E_{k}^{\left(2\right)},E_{k}^{\left(3\right)},E_{k}^{\left(4\right)},E_{k}^{\left(5\right)},E_{k}^{\left(6\right)}\right]}^{T}$) consists of the subjects’ extension state $x_{k}^{e,1}={\left[E_{k}^{\left(1\right)},E_{k}^{\left(2\right)},E_{k}^{\left(3\right)}\right]}^{T}$ and $x_{k}^{e,2}={\left[E_{k}^{\left(4\right)},E_{k}^{\left(5\right)},E_{k}^{\left(6\right)}\right]}^{T}$.

As mentioned in Section 3.2 and Section 3.3, the extension state is estimated by calculating the needed moments of $x_{k}^{e}$ and $P_{k}^{e}$ based on UT sampling, and implemented in the framework of Kalman filter (KF). The general KF has $O(N^{3})$ complexity in the number *N* of state and requires $O(N^{3})$ floating-point computations [32]. Thus, the computational complexity of our approach has the same order of magnitude as that of KF and mainly depends on the dimension of the selected extension state for tracking. As an example of an elliptical object, its computational complexity is $O(N^{3}).$ Since one elliptical object’s extension state is $x_{k}^{e,1}={\left[E_{k}^{\left(1\right)},E_{k}^{\left(2\right)},E_{k}^{\left(3\right)}\right]}^{T}$ with the dimension of 3, the computational complexity of a complex object is $O(N^{3}+{\left[3\left(N_{s}-1\right)\right]}^{3})$, where $N_{s}$ is the number of the decomposed elliptical sub-objects. Actually, $O(N^{3}+{\left[3\left(N_{s}-1\right)\right]}^{3})$ is equivalent to $O\left(N^{3}\right)+O\left({\left[3\left(N_{s}-1\right)\right]}^{3}\right)$ by neglecting the inconsequential high order items in practical applications. In other words, compared with the computational complexity of a single sub-object’s extension state estimation, the complex object’s computational complexity is added by $O({\left[3\left(N_{s}-1\right)\right]}^{3})$. The above will be analyzed and verified by the following experimental simulations in Section 4.3.

## 4. Simulation Results and Performance Evaluation

In this section, we focus on the case of a stationary sensor platform and moving extended object. Here, the proposed Minkowski-sum-based maneuvering modeling is applied to the MCEOT using range extent measurements, and the following three simulation scenarios of MCEOT are considered. In the first two scenarios, the proposed MCEO model is a constant turn maneuver model with known turn rates and its dynamic equation is linear. In the third scenario, it is a constant turn model with unknown turn rates and its dynamic equation is nonlinear.

To evaluate the tracking performance of the proposed approach, the following two approaches are compared by simulation to illustrate its effectiveness in these scenarios. To be fair for extension estimation, the compared approaches are initialized with the same extension (e.g., a circle) without further information:

(a) MCEOT-1: The proposed approach based on Minkowski sum considering the highly coupled dynamics of both the state and the extension.
(b) MCEOT-2: The approach considering only the centroid state dynamics.

MCEOT-2 is compared because there is rarely accounted for in existing literature to deal with the MCEOT of both centroid kinematics and the extension dynamics using target’s range extent. Thus, other techniques are not compared with the proposed approach (i.e., MCEOT-1) in simulations. However, to verify its effectiveness and benefit of considering the highly coupled dynamics of both the state and the extension for modeling and tracking, we compare MCEOT-1 with MCEOT-2 in the simulations.

The performance comparison results demonstrate that MCEOT-1 outperforms MCEOT-2, in which exchange of information between centroid kinematics and extension dynamics are sufficiently utilized to improve performance. The detailed discussions and analysis are as follows.

### 4.1. Tracking Performance Using Minkowski-Sum-Based CT Model with Known Rates

Consider the following two different scenarios in which an object with complex extension (as an example of Figure 1) performs a nearly constant velocity (CV) motion and two CT maneuvers in the 2D Cartesian coordinate system with the initial kinematic state $x_{0}^{m}=[1000\;$m,100m/s,2000m,60m/s${]}^{T}$. This object is composed and modeled by two elliptical sub-objects with different orientation angles using Minkowski sum, both of which have the same object extension (i.e., the lengths of minor and major axes are 10 m and 50 m, respectively). The sensor, fixed at the origin (0,0), provides measurements of range, bearing, down- and cross-range extent along line of sight every T=1 s. Each measurement is corrupted by zero-mean white Gaussian noise with standard deviations $\sigma_{r}=5m$, $\sigma_{\beta }=0.01$ rad, $\sigma_{D}=5m$, and $\sigma_{C}=5m$. These two scenarios are simulated to illustrate the effectiveness of our approach.

**Figure 3:**
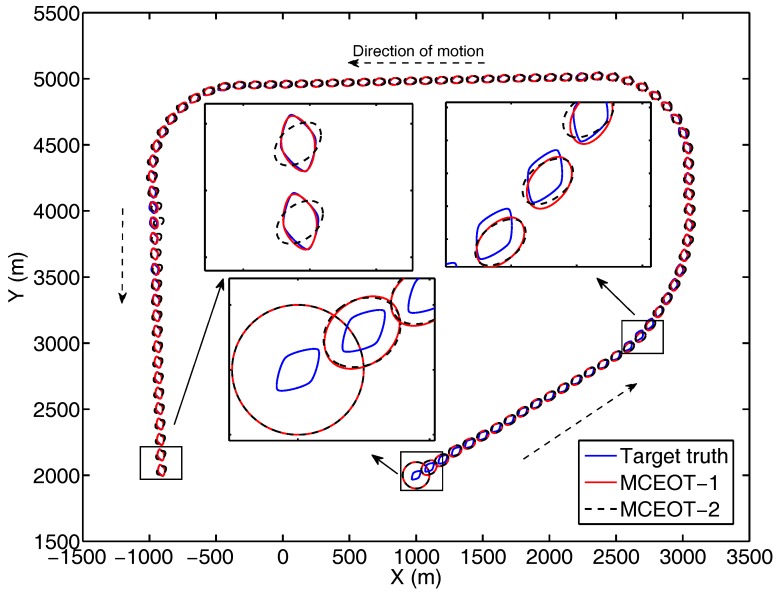
Trajectory of the complex extended object in scenario A (the blue solid line is for the true object, the red solid line and black dash line are for MCEOT-1 and MCEOT-2, respectively).

In the scenario A, the true trajectory is illustrated in Figure 3, i.e., this object undergoes two different turn maneuvers. The modal state of this scenario contains M=3 elements for the CV model and two CT models with turn rates $\omega_{A}^{\left(1\right)}=$5π/180 rad/s and $\omega_{A}^{\left(2\right)}=$ 10π/180 rad/s (it has a stronger maneuverability).

**Figure 4:**
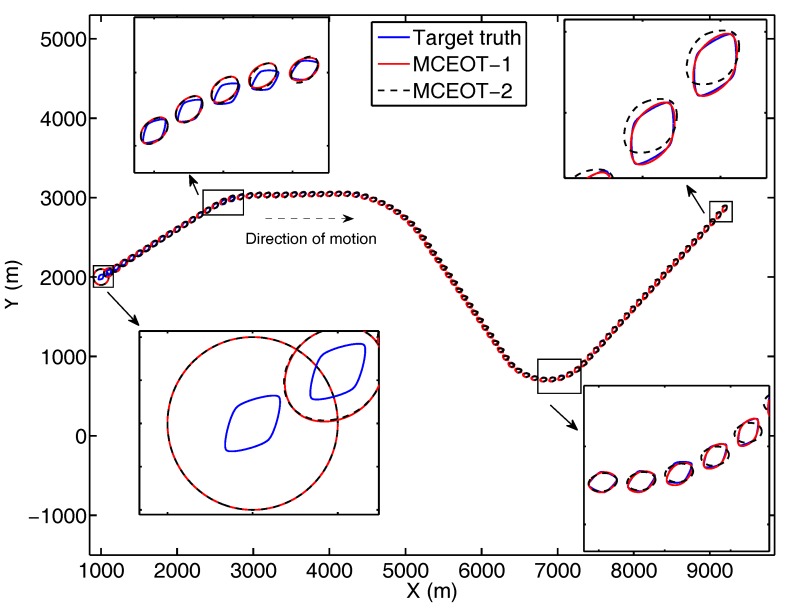
Trajectory of the complex extended object in scenario B (the blue solid line is for the true object, the red solid line and black dash line are for MCEOT-1 and MCEOT-2, respectively).

In scenario B, the true trajectory (illustrated in Figure 4) is more sophisticated, in which this object performs different turn motions in clockwise and anticlockwise order. The modal state of scenario B also contains M=3 elements for the CV model and two CT models with turn rates $\omega_{B}^{\left(1\right)}=$−5π/180 rad/s and $\omega_{B}^{\left(2\right)}=$10π/180 rad/s.

**Figure 5:**
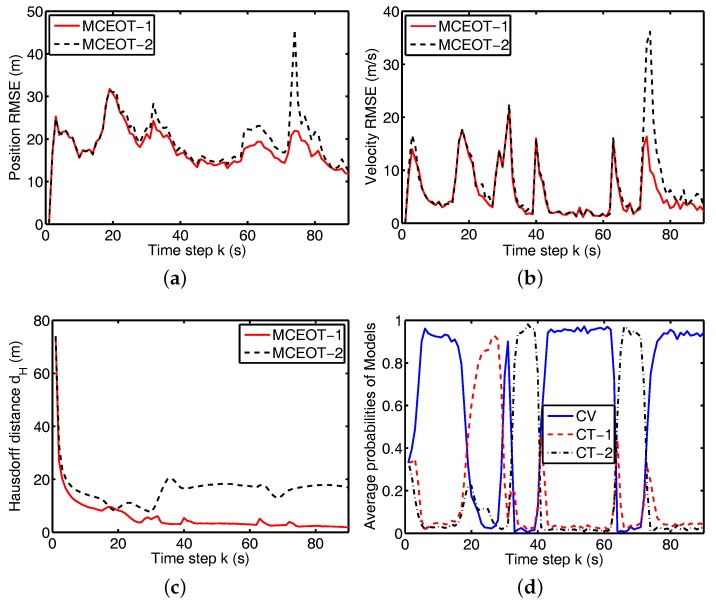
Performance comparison for scenario A. (**a**) position RMSE; (**b**) velocity RMSE; (**c**) Hausdorff distance; (**d**) average probability of MCEOT-1.

**Figure 6:**
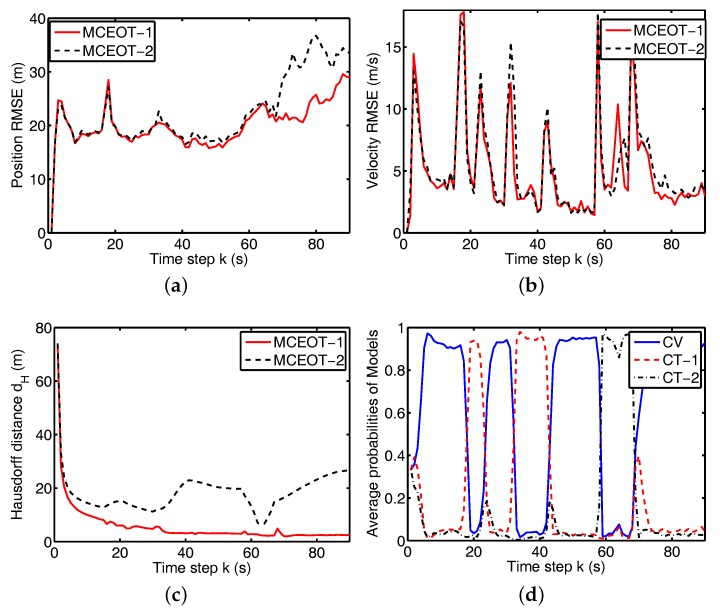
Performance comparison for scenario B. (**a**) position RMSE; (**b**) velocity RMSE; (**c**) Hausdorff distance; (**d**) average probability of MCEOT-1.

Simulation results for the above scenarios are shown in Figure 3, Figure 4, Figure 5 and Figure 6. The true and estimated object trajectories of different scenarios are shown in Figure 3 and Figure 4. In this simulation, the comparison results of kinematic state estimation are the root-mean-square error (RMSE) [33], i.e., it is chosen as the measure to evaluate performance of position and velocity estimation over 100 Monte Carlo runs (M=100). The RMSE of kinematics estimation has the following forms:

(34) $$\mathrm{RMSE}({\hat{x}}_{k}^{m})={\left(\frac{1}{M}\sum_{i=1}^{M}{∥{\tilde{x}}_{k,i}^{m}∥}^{2}\right)}^{1/2},$$

where ${\tilde{x}}_{k,i}^{m}=x_{k,i}^{m}-{\hat{x}}_{k,i}^{m}$ is the estimation error on the *i*th of the *M* Monte Carlo runs. The comparison results are shown in Figure 5a,b and Figure 6a,b. These figures show that MCEOT-1 has better performance than MCEOT-2 in estimation of kinematic state because MCEOT-2 considering only the state dynamics. It can be concluded that sufficiently utilizing extension dynamics information of the maneuvering extended object can effectively improve the accuracy of the kinematic state estimation. Actually, with the help of kinematic state estimation, the extension estimation can also be improved.

The object extension evaluation of an maneuvering extended object can be regarded as a problem of object extension matching; thus, the Hausdorff distance [34] is adopted to objectively measure the quality of extension estimation, which reflects the degree of similarity between the estimated extension and the true one. However, it is difficult to be used for performance evaluation of the extension estimation directly because the range of θ is a continuous set (i.e., θ∈[0,2π)). Thus, the Hausdorff distance is modified by

(35) $$d_{H}\left(K,\hat{K}\right)=\underset{\theta \in \{j\cdot \frac{2\pi }{N_{s}},j=1,\ldots N_{s}\}}{\frac{1}{M}\sum_{i=1}^{M}\sup\left|H_{K,i}\left(\theta \right)-H_{\hat{K},i}\left(\theta \right)\right|},$$

where $d_{H}\left(K,\hat{K}\right)$ is the modified Hausdorff distance between the estimated object extension $\hat{K}$ and the true one *K* on the *i*th of the *M* Monte Carlo runs, and θ∈[0,2π) is replaced by the discrete set $\theta \in \{j\cdot \frac{2\pi }{N_{s}},i=1,\ldots N_{s}\}$ via uniform angle sampling. In our simulation, $N_{s}$ as the number of sampling points is set to be 1000.

From Figure 3, Figure 4, Figure 5 and Figure 6, we can see that MCEOT-1 has almost the same performance as MCEOT-2 in the centroid kinematics and extension estimation when the extended object performs CV motion. However, MCEOT-1 outperforms MCEOT-2 (i.e., MCEOT-1 has smaller position and velocity RMSE, and the short Hausdorff distance) when this object maneuvers. In other words, MCEOT-1 estimates the kinematic state and object extension accurately while MCEOT-2 cannot (see Figure 5a–c and Figure 6a–c) because MCEOT-2 only focuses on centroid kinematics without considering of the object extension dynamics. Compared with MCOET-2, MCEOT-1 can produce simultaneously stable tracking and extension estimates converging to the true extended object.

As illustrated in Figure 5d and Figure 6d, MCEOT-1 distinguishes the true mode successfully and identifies the tracking model correctly matches the real situation, especially when the extended object starts or ends a maneuver.

### 4.2. Tracking Performance Using Minkowski-Sum-Based CT Model with Unknown Rates

**Figure 7:**
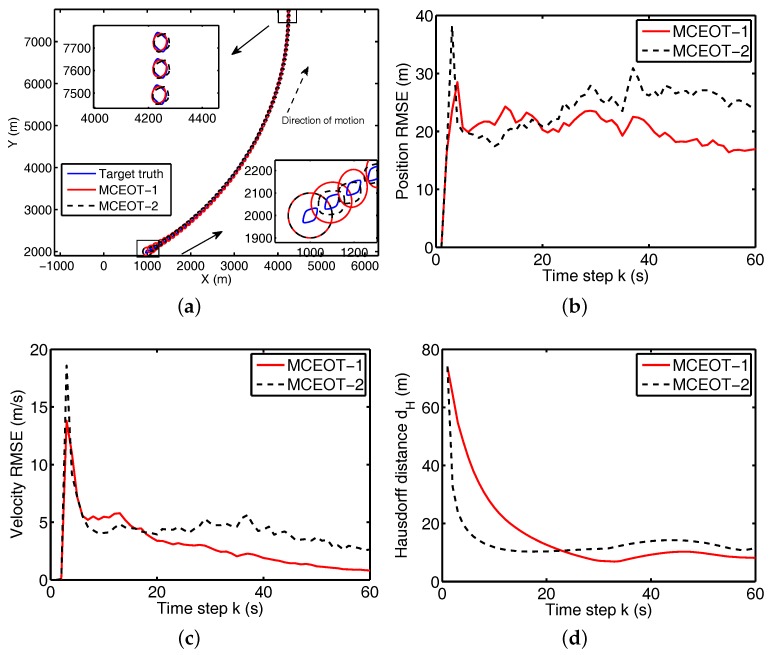
Simulation results in scenario C. (**a**) the object trajectory; (**b**) position RMSE; (**c**) velocity RMSE; (**d**) Hausdorff distance.

In the scenario C, the true trajectory is illustrated in Figure 7a: the extended object performs CT motion with turn rate $\omega_{C}=\pi /180$ from 1 s to 60 s. Unlike in Section 4.1, the turn rate $\omega_{C}$ of CT motion is totally unknown for the tracker in this scenario. Note that the other simulation parameters are the same as that of the extended objects in the Section 4.1. In addition, the compared approaches (i.e., MCEOT-1 and MCEOT-2) are also initialized with the same circle without further extension information for the fair evaluation.

From Figure 7b–d, we can see that simulation results and performance comparisons in this case are similar to that of Section 4.1, i.e., MCEOT-1 has better performance than MCEOT-2 in the centroid kinematics and extension estimation. Note that MCEOT-2 outperforms MCEOT-1 in the initial tracking process (roughly from 1 s to 20 s) because MCEOT-2 considers only the centroid state dynamics without extension dynamics, i.e., its dynamic model is:

(36) $$\begin{array}{c} x_{k}^{A}=F_{k-1}^{A}x_{k-1}^{A}+G_{k-1}^{A}w_{k-1}^{A}\\ \left[\begin{array}{c} x_{k}^{m}\\ e_{k}\\ \omega_{k} \end{array}\right]=\left[\begin{array}{ccc} F_{k-1}^{m} & 0 & 0\\ 0 & I_{6} & 0\\ 0 & 0 & 1 \end{array}\right]\left[\begin{array}{c} x_{k-1}^{m}\\ e_{k-1}\\ \omega_{k-1} \end{array}\right]+\left[\begin{array}{ccc} G^{m} & 0 & 0\\ 0 & I_{6} & 0\\ 0 & 0 & 1 \end{array}\right]\left[\begin{array}{c} w_{k-1}^{m}\\ w_{k-1}^{e}\\ w_{k-1}^{\omega } \end{array}\right], \end{array}$$

where $I_{6}=$ diag(1,1,1,1,1,1). This dynamic model is really nonlinear because this unknown turn rate is considered as a unknown state component and augmented by the joint state. However, compared with the dynamic model of Equation (28), its nonlinearity is weaker for tracking. Thus, MCEOT-2 achieves better performance than MCEOT-1 in the initial period. As time goes on, however, MCEOT-1 beats MCEOT-2 in the final steady state (see Figure 7). This main reason is that MCEOT-1 considers the highly coupled dynamics of both the state and the extension. The estimation of the kinematic state and extension are inter-dependent and they affect each other. In this case, more and more two-way exchange of information between centroid kinematics and extension dynamics is sufficiently utilized by MCEOT-1 to achieve much better estimation performance than MCEOT-2.

The above simulation results demonstrate that good solutions to the MCEOT problem need to fully consider the highly coupled dynamics of both the state and the extension. It can be concluded that the validity of the proposed approach (MCEOT-1) is verified by simulation results and performance comparison in different scenarios.

### 4.3. Performance Comparison and Complexity Analysis

To verify the complexity analysis of Section 3.4 in the experimental aspect, the following approach is compared with MCEOT-1 in scenario A.

MEOT-1: The elliptical maneuvering extended object modeling and tracking approach.

Note that the simulation parameters are the same as that of the Section 4.1, and the compared approaches are still initialized with the same extension (e.g., a circle) without further information. The computational complexities of the MCEOT-1 and the MEOT-1 are analyzed and compared as follows. Specifically for MCEOT-1 and MEOT-1, their computational complexities are reflected by the time consumption in simulations. The most time-consuming parts of the above approaches lie in the estimation of the object extension state. MCOET-1 has more complexity mainly because it requires more computational time than MEOT-1 for estimating the extension state with higher dimension. Table 1 shows the one-run computational time of the compared approaches for scenarios A and B averaged over the 100 Monte Carlo runs. In the simulation experiments, the above two approaches are implemented in the Matlab (R2011b) environment on a computer with a 4.00 GHz CPU (Intel Core i7 4790k), only a single thread is used). Clearly from this table, MCOET-1 requires more computational time for tracking (aiming at estimating $x_{k}^{e}={\left[{\left(x_{k}^{e,A}\right)}^{T},{\left(x_{k}^{e,B}\right)}^{T}\right]}^{T}={\left[E_{k}^{\left(1\right)},E_{k}^{\left(2\right)},E_{k}^{\left(3\right)},E_{k}^{\left(4\right)},E_{k}^{\left(5\right)},E_{k}^{\left(6\right)}\right]}^{T}$) than MEOT-1 (aiming at estimating $x_{k}^{e,A}={\left[E_{k}^{\left(1\right)},E_{k}^{\left(2\right)},E_{k}^{\left(3\right)}\right]}^{T})$. The results of Table 1 are consistent with the complexity analysis in Section 3.4.

**Figure 8:**
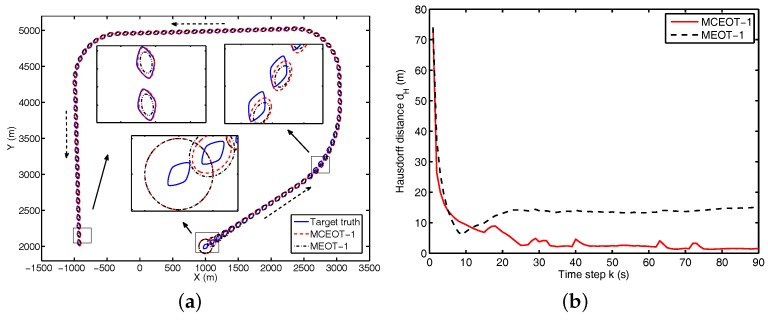
Simulation results in scenario A. (**a**) the object trajectory; (**b**) Hausdorff distance.

As shown in Figure 8, MCEOT-1 outperforms MEOT-1 in estimation of extension state for scenario A. The main reason is that using the simple elliptical extension dynamics model for tracking of a complex object ignores more detailed shape information. This does not facilitate the improvement of estimation performance. Specially during the extended object maneuvers, MCEOT-1 can estimate the object extension accurately while MEOT-1 cannot because the MEOT-1 does not consider the actual extension maneuvers and uses an inappropriate model. As time goes by, more and more tracking errors are cumulated, which go against the accurate estimation of a maneuvering complex extended object. Correspondingly, it achieves much weaker performance than MCEOT-1 in the final steady state (see Figure 8), though it takes less time.

In summary, for both MCEOT-1 and MEOT-1, their computational complexities mainly differ in the estimation of the object extension state, which are reflected by the time-consumption of tracking. Compared with MEOT-1, MCEOT-1 gets much better results and its time complexity is of the same order. Due to the concise linear form, the proposed approach can achieve excellent tracking performance with the rise of low complexity.

## 5. Conclusions

To deal with MCEOT using range extent measurements, this paper proposed a general approach based on Minkowski sum. It not only accurately describes different turn maneuvers in both the extension dynamics and the centroid kinematics, but also fully considers the close coupling between the centroid maneuver and the abrupt change of extension. Thank to its concise and unified form, the complex extension dynamics can be easily described by several sub-objects’ extension dynamics in a joint way. This largely simplifies the whole modeling process of an MCEO. Furthermore, the proposed Minkowski-sum-based modeling is simple and effective, and it can be easily implemented to deal with different cases of the turn maneuvers with known/unknown turn rates for MCEOT. The effectiveness of what we proposed is demonstrated through simulation results, which achieves excellent performance in the estimation of the centroid kinematic state and the object extension jointly with low complexity.

Furthermore, the practical benefits of our approach are summarized as follows: (1) due to its concise mathematical forms and favorable properties, the maneuvering complex extended object modeling is largely simplified and more detailed shape information is described; (2) a larger range of maneuvering complex extended object tracking can be handled by using Minkowski sum. In summary, our approach may pave the way for solving different MCEOT using range extent measurements.

## Figures and Tables

**Table 1:** Averaged computation time (seconds) for one run (90 steps) of two approaches in scenario A.

MCOET-1	MEOT-1
0.2971	0.1103
